# Combined inhibition of MET and VEGF enhances therapeutic efficacy of EGFR TKIs in *EGFR*-mutant non-small cell lung cancer with concomitant aberrant MET activation

**DOI:** 10.1186/s40164-024-00565-9

**Published:** 2024-10-01

**Authors:** Shanshan Huang, Yaling Long, Yuan Gao, Wanling Lin, Lei Wang, Jizong Jiang, Xun Yuan, Yuan Chen, Peng Zhang, Qian Chu

**Affiliations:** Department of Oncology, Tongji Hospital, Tongji Medical College, Huazhong University of Science and Technology, Wuhan, China

**Keywords:** MET, Non-small cell lung cancer, VEGF/VEGFR2 signaling, EGFR TKIs resistance

## Abstract

**Background:**

Aberrant activation of mesenchymal epithelial transition (MET) has been considered to mediate primary and acquired resistance to epidermal growth factor receptor (EGFR) tyrosine kinase inhibitors (TKIs) in *EGFR*-mutant non-small cell lung cancer (NSCLC). However, mechanisms underlying this process are not wholly clear and the effective therapeutic strategy remains to be determined.

**Methods:**

The gefitinib-resistant NSCLC cell lines were induced by concentration increase method in vitro. Western blot and qPCR were used to investigate the relationship between MET and vascular endothelial growth factor (VEGF)/VEGF receptor 2 (VEGFR2) signaling pathway. Double luciferase reporter gene and co-immunoprecipitation were used to further reveal the regulation mechanism between MET and VEGF/VEGFR2. The effect of combined inhibition of MET and VEGF/VEGFR2 signaling pathway on the therapeutic sensitivity of EGFR-TKI in gefitinib resistant cell lines with MET aberration was verified ex vivo and in vivo.

**Results:**

We successfully obtained two gefitinib-resistant NSCLC cell lines with *EGFR* mutation and abnormal activation of MET. We observed that MET formed a positive feedback loop with the VEGF/VEGFR2 signaling, leading to persistent downstream signaling activation. Specifically, MET up-regulated VEGFR2 expression in a MAPK/ERK/ETS1-dependent manner, while VEGF promoted physical interaction between VEGFR2 and MET, thereby facilitating MET phosphorylation. A MET inhibitor, crizotinib, combined with an anti-VEGF antibody, bevacizumab, enhanced the sensitivity of NSCLC cells to gefitinib and synergistically inhibited the activation of downstream signaling in vitro. Dual inhibition of MET and VEGF combined with EGFR TKIs markedly restrained tumor growth in both human NSCLC xenograft models and in an *EGFR*/*MET* co-altered case.

**Conclusions:**

Our work reveals a positive feedback loop between MET and VEGF/VEGFR2, resulting in continuous downstream signal activation. Combined inhibition of MET and VEGF/VEGFR2 signaling pathway may be beneficial for reversing EGFR TKIs resistance.

**Supplementary Information:**

The online version contains supplementary material available at 10.1186/s40164-024-00565-9.

## Background

Lung cancer is the leading cause of cancer death worldwide [1]. Non-small cell lung cancer (NSCLC) is the most common histological type of lung cancer, accounting for approximately 85% of all lung cancer cases, including over 50% of lung adenocarcinomas and 30% of lung squamous cell carcinomas [2]. Early NSCLC is typically asymptomatic, and most patients are diagnosed at stage III or IV, often lacking surgical indications and with poor prognosis. Over the past few decades, the development of molecule-targeted therapies based on oncogenic driver mutations has significantly improved the overall survival and quality of life of patients with actionable driver mutations [3]. *EGFR*-activating mutations, which occur in nearly 10% of NSCLCs in Caucasians and up to 50% in Asians, have been identified as driver oncogenes for NSCLC [4–6]. Although most *EGFR*-mutant NSCLC patients exhibit a high clinical response rate to EGFR TKIs, a majority experience therapy failure due to acquired resistance within 1 ~ 2 years. Approximately 20 ~ 30% of patients receiving EGFR TKIs show primary drug resistance and rapid progress [7–10]. To date, primary and acquired resistance to EGFR TKIs, via EGFR-dependent as well as EGFR-independent mechanisms, remains a big challenge in the management of *EGFR*-mutant NSCLC. Aberrant activation of mesenchymal epithelial transition factor (MET) signaling is recognized as a mechanism that underlies EGFR-independent resistance to EGFR TKIs [11–14].

MET, also known as the hepatocyte growth factor receptor (HGFR), is a membrane-bound receptor tyrosine kinase (RTK). The extracellular portion of MET contains of the semaphorin (Sema) domain, PSI domain, and the immunoglobulin–plexin–transcription (IPT) domain. Intracellularly, MET contains a tyrosine kinase catalytic domain flanked by distinctive juxtamembrane and carboxy-terminal docking site. This portion of MET contains the catalytic tyrosines, Y1234 and Y1235, which positively modulate enzyme activity [15]. MET signaling may become dysregulated or aberrant through several mechanisms, including the overexpression of MET protein or *MET* gene alterations, such as mutations, amplification, or rearrangement. *MET* amplification is one of the common causes of bypass pathway activation among the resistance mechanism of EGFR TKIs [16]. Acquired *MET* amplification accounts for 5–24% of the EGFR TKI-resistant cases, while de novo *MET* amplification accounts for approximately 3% of *EGFR*-mutated NSCLC patients before treatment [17, 18]. Studies have shown that *MET* amplification induces autophosphorylation of MET protein and which associates with ERBB3 to activate the PI3K/AKT pathway, leading to the development of resistance to EGFR TKIs [19]. Although some studies have indicated that other MET aberrations which activate the MET pathway may also confer resistance to EGFR TKIs, the incidence of these cases is not wholly clear [20]. Thus, MET is a potentially attractive therapeutic target in the treatment of patients with EGFR TKI-resistant NSCLC. Currently, major efforts are being made to detect effective treatment strategies to overcome MET aberrant-mediated EGFR TKIs resistance. However, the potential efficacy of combination therapy strategies as a standard of care for these patients may need further evaluation [21, 22].

Tumor angiogenesis plays an important role in the process of tumor growth and metastasis, and anti-tumor angiogenesis drugs have been widely used in clinical practice. Among the many factors known to promote angiogenesis, vascular endothelial growth factor (VEGF) is one of the most potent pro-angiogenic factors. VEGF-binding induces conformational changes in VEGFRs followed by receptor dimerization and autophosphorylation of tyrosine residues in the intracellular kinase domains, leading to alterations in cell proliferation, migration, differentiation, and tube formation [23]. The role of VEGF in tumor angiogenesis via stimulation of VEGFRs on tumor endothelium is well established. However, evidence is emerging that VEGF may play an additional role in cancer through stimulation of VEGFRs on tumors cells. Furthermore, several experimental tumor models have indicated that tumor cell-specific VEGFRs expression acts as a critical driver in the pathogenesis of tumors. VEGF/VEGFRs inhibition results in a reduced tumor burden, not only by impairing angiogenesis but also by attenuating tumor cell proliferation [24, 25].

Bevacizumab, a humanized monoclonal antibody targeting VEGF, has been shown to significantly prolong progression-free survival (PFS) in advanced NSCLC when administered in combination with standard first-line platinum-based chemotherapies or EGFR TKIs [26]. However, the efficacy of additional treatment with bevacizumab for the subgroup of *EGFR*-mutant NSCLCs with aberrant MET activation remains unknown. Evidence increasingly indicates that the distinct associations between VEGFRs and other RTKs may reflect a level of regulation via which VEGF is able to induce positive or negative signaling in different cell types [27]. In addition, simultaneous activation of MET and VEGF signaling results in cooperative up-regulation of many different molecular pathways, leading to more robust proliferative responses [28]. Therefore, we attempted to explore the mutual regulatory relationship between MET and VEGF signal transduction in NSCLC, in order to further clarify the potential value of combined inhibition of MET and VEGF on enhancing sensitivity to EGFR TKIs in *EGFR*-mutant NSCLCs with aberrant MET activation.

Here, we successfully obtained two gefitinib-resistant NSCLC cell lines with *EGFR* mutation and abnormal activation of MET. Moreover, we detected a positive feedback interaction between MET and VEGF/VEGFR2 signaling. Specifically, MET up-regulated VEGFR2 expression in a MAPK/ERK/ETS1-dependent manner, while VEGF promoted physical interaction between VEGFR2 and MET, thereby facilitating MET phosphorylation. The activation of this positive feedback loop increased the sensitivity of cells to VEGF, thus promoting the activation of downstream signals. Most importantly, combined inhibition of MET and VEGF enhanced the antitumor activity of EGFR TKIs in vitro and in vivo. Collectively, our study presents a promising therapeutic strategy for *EGFR*-mutant NSCLC with concomitant aberrant MET activation.

## Methods

### Cell cultures and reagents

Human NSCLC cell lines (H441, H1299, A549, PC-9, HCC827 and H1975) and a normal bronchial epithelial cell (BEAS-2B) were obtained from the oncology laboratory of Tongji Hospital, Wuhan, China. These cells were cultured in RPMI-1640 medium (HyClone, Provo, UT) supplemented with 10% fetal bovine serum (FBS; Gibco, MD, USA). The HEK-293T cells (obtained from the oncology laboratory of Tongji Hospital) were cultured in DMEM (HyClone, Logan, UT) supplemented with 10% FBS. All cells were cultured in a humidified incubator under conditions of 37 °C and 5% CO2, and no mycoplasma contamination was found. Recombinant VEGF was purchased from PeproTech (Rocky Hill, NJ). Osimertinib, U0126, LY294002, Stattic, SCH772984, apatinib and vandetanib was purchased from MedChemExpress (Monmouth Junction, NJ). Gefitinib and crizotinib was purchased from Selleck (Shanghai, China). Bevacizumab was obtained from Roche Pharma. All products were stored and used according to the manufacturer’s instructions.

## Cell counting kit-8 (CCK8) assay

Cells were plated into 96-well plates at a density of 2 × 10^3^ cells/100 µl RPMI-1640 medium with 10% FBS per well. After 24-hour incubation, various reagents were added to each well, and the cells incubated for a further 48 h, followed by the addition of 100 μl 10% CCK8 solutions (Promotor, Wuhan, China) to each well and incubation for 2 h. Cell viability was examined by measuring the absorbance at 450 nm via a Power Wave XS microplate reader (BioTek, VT, USA). Each reagent concentration was tested at least in triplicate during each experiment, and each experiment was conducted at least three times.

## Western blotting and co-immunoprecipitation (co-IP) analysis

Cells were washed three times with ice-cold PBS and lysed by RIPA lysis buffer (Beyotime, Shanghai, China) containing phenylmethylsulphonyl fluoride (PMSF) and phosphatase inhibitors (Promotor, Wuhan, China) for 30 min on ice. Then, the whole‐cell lysates were centrifuged at 4 °C, 12 000 g for 20 min and the protein was collected. The protein concentration was measured by the BCA protein assay kit (Beyotime, Shanghai, China). Equal amounts of denatured proteins were separated by 10% SDS‐PAGE gels and transferred to PVDF membranes (Millipore, Massachusetts, USA). The membranes were blocked with 5% skim milk or BSA for 1 h at room temperature. Primary antibodies were added, and the membrane was incubated at 4 ℃ overnight, then incubated with corresponding secondary antibodies (Promotor, Wuhan, China) for 1 h at room temperature. Finally, the immunoblots were detected by SuperSignal West Pico Chemiluminescent Substrate (Thermo Fisher, USA). Images were captured with SynGene G: Box Chemi XRQ (Alpha Metrix Biotech, Rödermark, Hesse, Germany), and intensity of blot bands was analyzed by ImageJ 1.8.0 (National Institutes of Health, Bethesda, MA, USA).

For immunoprecipitation (IP) experiments, cells were washed in cold PBS, lysed in NP-40 (Beyotime) containing PMSF and phosphatase inhibitors for 30 min and then centrifuged at 12 000 g for 20 min. The cell lysates were incubated with 1 µg of antibodies overnight at 4 °C and then incubated with 30 µL of protein A/G magnetic beads (MedChemExpress, NJ) for 2 h at 4 °C. After washing and isolating the beads with a magnet, the beads were boiled for 10 min in SDS protein loading buffer to elute the bound protein. Subsequently, the IP lysates were assessed by western blotting analysis with the indicated antibodies. The primary antibodies used are enlisted in the Supplemental Table S1.

## Quantitative real-time PCR (qPCR)

Total RNA was extracted from cells using TRIzol reagent (Takara, Japan) and reverse transcribed using a PrimeScript RT-PCR kit (Takara, Japan) according to the manufacturer’s instructions. Real-time PCR analysis was performed using SYBR Premix Ex Taq (Takara, Japan) with a 7900 Real-time PCR system (Applied Biosystems, USA). β-actin was used as an internal control. The relative gene expression levels were calculated using the comparative cycle threshold method, in which the relative expression is calculated as 2ΔΔCt. Three replicate PCR amplifications were performed for each sample. The sequences of the primers are shown in Supplemental Table S2.

## Lentivirus transduction

Cell culture and transfection were performed as previously described [29]. Cells were cultured for 24 h to 60% confluence in complete medium containing 10% FBS before transfection. Subsequently, the cell culture medium was replaced with serum-free medium containing lentiviruses (GeneChem, Shanghai, China) at a multiplicity of infection of 20 and polybrene (GeneChem) at a final concentration 5 µg/ml. Cells as a negative control were transduced with corresponding empty vector lentiviruses in the same manner. After 12 h, the cell culture medium was changed to complete medium for another 48 h. Then cells were cultured in culture medium containing 2 µg/ml puromycin for 1 week to select stably transduced cells. The expression level of MET was evaluated by western blotting and qPCR.

### Luciferase activity assay

The luciferase reporter plasmids containing pGL3-basic-VEGFR2-promoter sequence were constructed by WZ Biosciences Inc. (Shandong, China). Luciferase reporter assays were performed as described in the manufacturer’s protocol. Both transductions and transfections were carried out in triplicate, and each experiment was repeated at least twice. 48 h later, cells were harvested and assayed using Dual-Luciferase Reporter Assay System (Promega, USA). Absorbance ratios of Firefly to Renilla luciferases were normalized to the control.

## Animal experiments

Five-week‐old NCG mice were used for the construction of the xenograft model in vivo. Cells (5 × 10^6^/100 µl PBS) were inoculated subcutaneously into right flanks of each mouse. When tumor volumes reached about 200 mm^3^, the mice were randomly divided into flowing groups with different combinations with or without bevacizumab (100 µg/mouse, intra-peritonelly), gefitinib (10 mg/kg, intragastrically), crizotinib (25 mg/kg, intragastrically). Each tumor was measured in two dimensions every 3 days and the volume was calculated using the following formula: (length (mm) × (width (mm))^2^)/2. After 2 weeks of administration, the mice were sacrificed, and the tumor tissues were fixed and analyzed by immunohistochemical staining.

## Tissue immunohistochemistry and immunofluorescence staining

Immunohistochemical staining based on the two-step standard IHC protocol was conducted as previously described [30]. The tumors were excised at the end of xenograft experiment, before being fixed in 4% paraformaldehyde and embedded in paraffin. Immunohistochemistry and immunofluorescence staining were conducted following standard procedures. Primary antibodies used for immunohistochemistry included: Ki-67 (1:200, #9027, Cell Signaling Technology, MA, USA). Primary antibodies for immunofluorescence included: CD31 (1:100, ab28364, Abcam, UK). Apoptotic cells were determined by the TUNEL assay according to the manufacturer’s protocols (Vazyme, China).

### Statistical analysis

Chart generation and statistical calculations were performed using the GraphPad Prism software (v8.0; CA, USA). All descriptive statistics were presented as the mean ± standard deviation (SD). Statistical comparisons between two experimental groups were performed using Student’s t test. If multiple groups were compared, the one-way analysis of variance (ANOVA) was performed. The Pearson’s correlation was used to measure the strength of association between two variables. A *P* value less than 0.05 was considered significant (*P < 0.05, **P < 0.01, ***P < 0.001).

## Results

### Construction and identification of gefitinib resistant *EGFR*-mutant NSCLC with concomitant aberrant MET activation

**Fig. 1:**
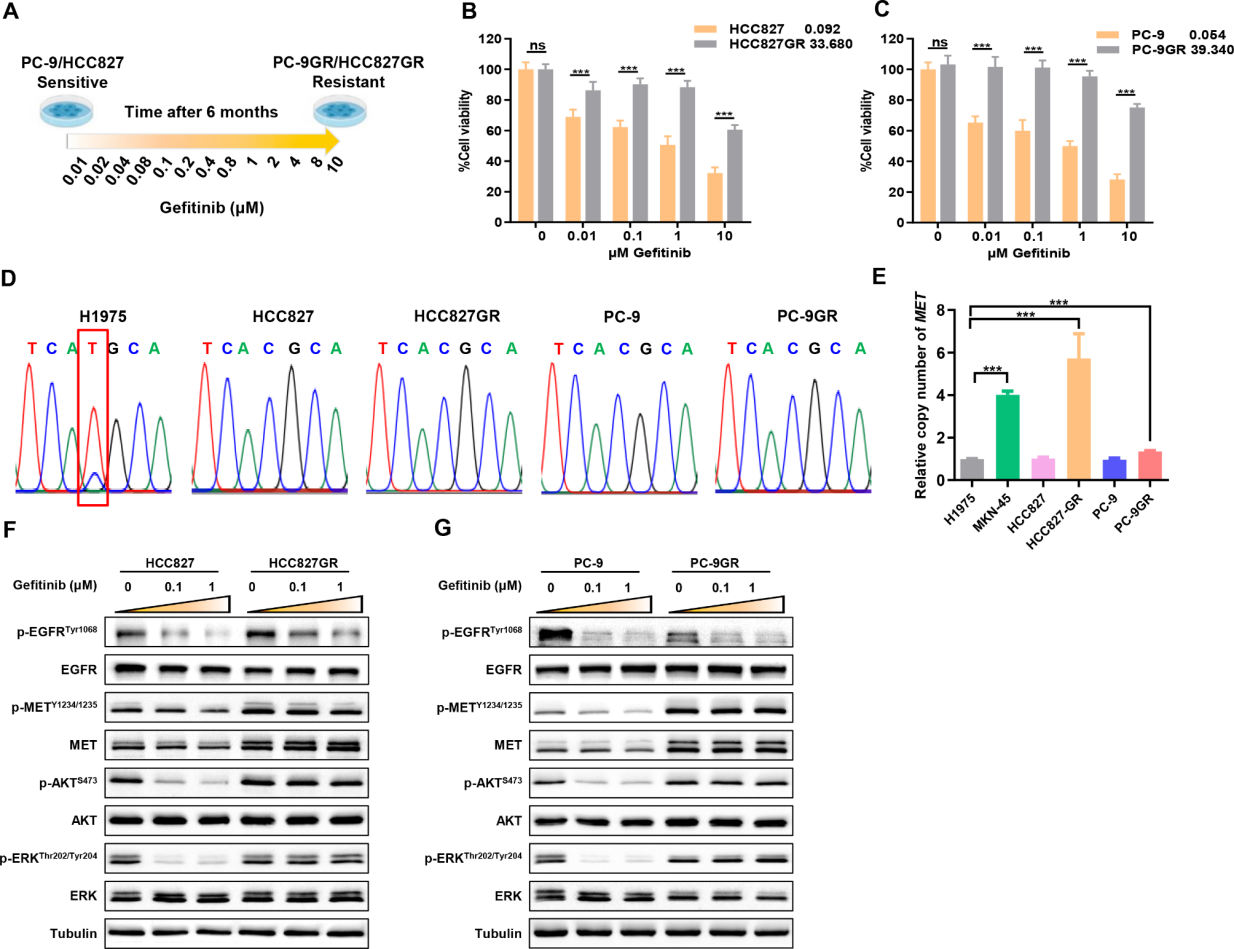
Construction and identification of gefitinib resistant *EGFR*-mutant NSCLC with concomitant aberrant MET activation (**A**) Experimental design to generate the gefitinib-resistant cell clones. (**B**) (**C**) Cells treated with increasing concentrations of gefitinib. Cell viability relative to untreated controls was measured by CCK-8 assays after 48 h. (**D**) DNA sequencing peaks of the cell line shown, with codon changes involving the *EGFR T790M* sequence marked in red boxes (ACG > ATG). (**E**) Relative quantitative analysis of copy number of *MET* gene in the indicated cells. (**F**) (**G**) Western blotting analysis of the expression of related signaling molecules in gefitinib-resistant cells and their parent cells. *P < 0.05, **P < 0.01, ***P < 0.001 (Student’s *t* test.). Graphs show mean ± SD

To explore the underlying molecular mechanism of MET-mediated EGFR TKIs resistance and potential therapeutic strategies, we established and screened gefitinib acquired resistance NSCLC cell lines. Lung adenocarcinoma cell lines HCC827 and PC-9 carrying an activating small in-frame deletion in exon 19 of EGFR (EGFR 19del) showed sensitivity to gefitinib (Supplemental Figure S1A, S1B). The gefitinib-resistant PC-9GR cells were induced by concentration increase method in *vitro*(Fig. 1A**).** Western blot was used to screen the clones of drug resistant strains with high expression and abnormal activation of MET (Supplemental Figure S1C). HCC827GR cells with *MET* amplification and aberrant MET activation was a gift from professor Zhu [31]. Increased gefitinib IC50 was observed in HCC827GR and PC-9GR cells (Fig. 1B and C). As expected, bypass activation of MET also resulted in HCC827GR and PC-9GR cells being resistant to the third-generation EGFR-TKI osimertinib (Supplemental Figure S1D, S1E). Both of HCC827GR and PC-9GR cells had no T790M mutation but *MET* gene copy number of were higher than that in their parental cells (Fig. 1D and E). Unlike in the parental cells, phosphorylation of ERK and AKT were maintained at relatively high levels in the HCC827GR and PC-9GR cells treated with gefitinib (Fig. 1F and G). These data confirmed the emergence of TKI resistance and aberrant MET activation in HCC827GR and PC-9GR cells.

### MET up-regulates VEGFR2 expression in a MAPK/ERK/ETS1-dependent manner in gefitinib resistant NSCLC

**Fig. 2:**
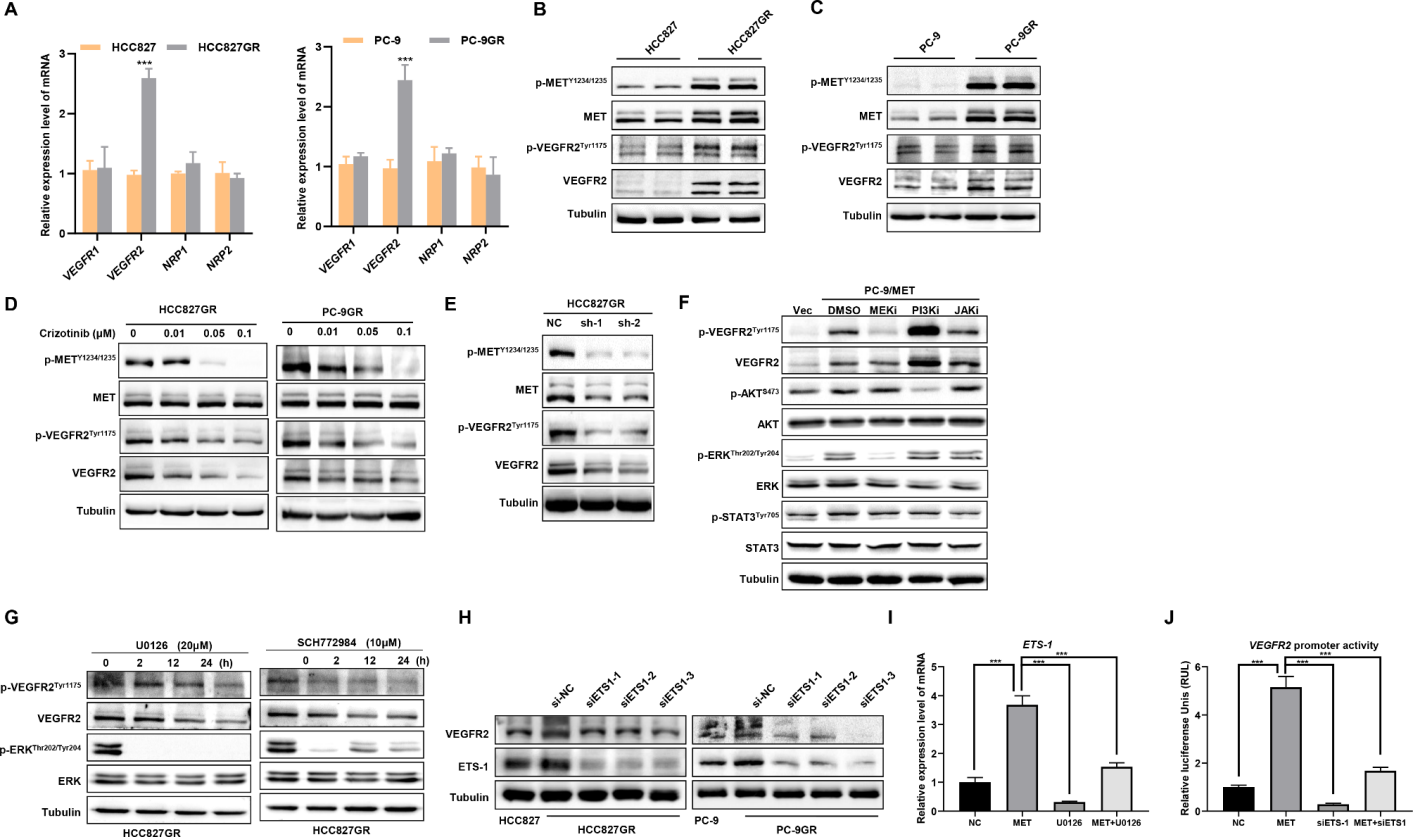
MET up-regulates VEGFR2 expression in a MAPK/ERK/ETS1 dependent manner (**A**) qPCR analysis of the expression of VEGF-associated receptor or co-receptor in indicated cells. (**B**) (**C**) Western blotting analysis of the expression of MET, VEGFR2 and their phosphorylation levels in indicated cells. (**D**) Western blotting analysis of the expression of MET, VEGFR2 and their phosphorylation levels indicated cells upon treatment with crizotinib at different concentrations for 6 h. (**E**) Western blotting analysis of the expression of MET, VEGFR2 and their phosphorylation levels in HCC827GR cells subjected to knockdown of MET (shMET). (**F**) Western blotting analysis of the expression and phosphorylation of VEGFR2 in PC-9/MET cells upon treatment with DMSO, MEK, PI3K, or JAK small molecular inhibitor for 24 h compared with PC-9/Vec cells. (**G**) Western blotting analysis of the expression and phosphorylation of VEGFR2 in HCC827GR cells upon treatment with U0126 or SCH772984. (**H**) Western blotting analysis of the expression of ETS1 and VEGFR2 in HCC827GR and PC-9GR cells transfected with ETS1 siRNA. (**I**) *ETS1* mRNA levels were monitored in PC-9 cells transfected with MET alone or treated with U0126. (**J**) Dual Luciferase reporter assay was performed to check the promoter activities of VEGFR2 in PC-9 cells transfected with MET alone or ETS1-siRNA. *P < 0.05, **P < 0.01, ***P < 0.001 (Student’s *t* test.). Graphs show mean ± SD

Anti-angiogenesis drugs targeting VEGF and its receptors have been recognized as an effective anti-tumor treatment strategy. In order to determine whether MET regulates the transmission of VEGF signaling in gefitinib resistant *EGFR*-mutant NSCLC, we examined the effect of MET overactivation on the expression of VEGF-associated receptors or co-receptors. Intriguingly, we found a significant increase of *VEGFR2* mRNA level in *MET*- aberrant cell lines (Fig. 2A). Next, we compared the level of VEGFR2 protein in gefitinib resistant *EGFR*-mutant cells with aberrant MET to that in the parental cells by western blotting. Notably, MET overactivation induced a visually noticeable increase in the protein expression of VEGFR2 in HCC827GR and PC-9GR cell lines (Fig. 2B and C).

To further clarify that the abnormal activation of MET signaling is directly related to the increased expression of VEGFR2. HCC827GR cells and PC-9GR cells were treated with crizotinib at different concentrations. After 6 h, the expression and phosphorylation activity of MET and VEGFR2 were detected. The results showed that crizotinib significantly inhibited MET phosphorylation activity in both cell lines, but had no significant effect on the background expression of MET protein, and crizotinib significantly inhibited VEGFR2 expression and phosphorylation activity in a dose-dependent manner (Fig. 2D). As shown in Fig. 2E, shRNA-mediated downregulation of MET expression also decreased MET phosphorylation in HCC827GR cells. At the same time, the expression level and phosphorylation activity of VEGFR2 in HCC827GR cells were significantly decreased. These results suggest that activation of MET signaling is involved in the regulation of VEGFR2 expression and activity. Next, we compared the level of VEGFR2 protein in *MET*-transfected cells to that in the vector control cells by western blotting. Notably, MET overactivation induced a visually noticeable increase in the protein expression of VEGFR2 in PC-9 and H1299 cell lines. In addition, HEK293T cells transfected with different qualities of *MET* plasmid also showed dose-dependent up-regulation of VEGFR2 expression compared with that in parental cells (Supplemental Figure S2).

Transcriptional control of VEGFR2 expression is complex and realized by multiple mechanisms. MET, is known to activate several intracellular signaling pathways, including MAPK, phosphoinositide 3-kinase (PI3K), and STAT3 [32]. We investigated whether any of these pathways were involved in regulating VEGFR2 expression. PC-9/MET cells were pretreated with several specific kinase inhibitors for 24 h. Consistent with previous reports, *MET*-transfected cells showed higher p-MET levels followed by the activation of downstream targets of ERK, AKT, and STAT3. U0126 (which is inhibitor of MEK1, the activator of MAPK) blocked the activation of ERK, LY294002, an inhibitor of PI3K, blocked the activation of AKT, and Stattic blocked STAT3 activation. We found that the up-regulation of VEGFR2 by MET was blocked by U0126 but not by LY294002 or Stattic (Fig. 2F). Meanwhile, VEGFR2 was downregulated in a time-dependent manner in HCC827GR cells upon treatment with U0126 and SCH772984 (a selective ERK inhibitor) (Fig. 2G).

P-ERK is known to regulate a variety of transcription factors, among which p-ERK-induced ETS1 expression and nuclear translocation have been reported to promote VEGFR2 transcription by binding to VEGFR2 promoter in bovine and mouse endothelial cells. We sought to test the existence of such transcriptional regulation in human NSCLC cell lines. As shown in Fig. 2H, the Western blot analysis demonstrated that *ETS1* knockdown led to the downregulation of VEGFR2 in HCC827GR and PC-9GR cells. Furthermore, we examined ETS1 expression in PC-9/MET cell lines with MET overexpression, and the results showed that MET significantly promoted ETS1 expression, and treatment with U0126, a MEK/ERK signaling pathway inhibitor, significantly down-regulated ETS1 expression (Fig. 2I). Prediction analysis of transcription factors by PROMO database and GeneCards database also showed the presence of ETS1 binding sequence in the promoter region of human VEGFR2. We synthesized a 2.3-kb sequence upstream of the first exon of VEGFR2 into a pGL3-Basic luciferase reporter vector to detect the activity of the VEGFR2 promoter in PC-9 cells treated with different co-transfection systems. The results showed that overexpression of MET in PC-9 cells increased the promoter activity of VEGFR2 by about 5-fold, while interference with ETS1 expression (transfection of si-ETS1) significantly inhibited the promoter activity of VEGFR2. In addition, interference with ETS1 expression in MET-overexpressing cells also significantly inhibited MET-promoted VEGFR2 transcriptional activity (Fig. 2J). These results indicated that MET-mediated up-regulation of VEGFR2 may be dependent on the MAPK/ERK/ETS1 pathway.

### VEGF induces MET activation through VEGFR2/MET complex in NSCLC cells

**Fig. 3:**
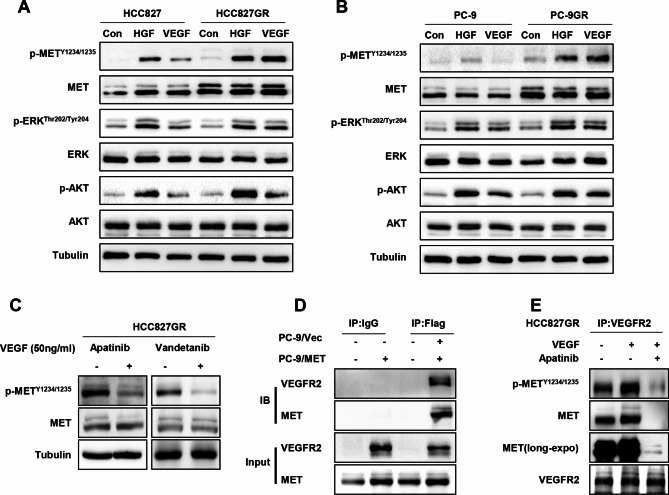
VEGF induces MET activation through VEGFR2/MET complex in NSCLC cells (**A**) (**B**) Western blotting analysis of the expression of the indicated proteins and their phosphorylation levels in indicated cells upon treatment with VEGF (50ng/ml) for different time. (**C**) Western blotting analysis of the expression of the indicated proteins and their phosphorylation levels in HCC827GR cells upon treatment with the VEGFR2 inhibitors apatinib or vandetanib for 24 h. (**D**) (**E**) Immunoprecipitation assay of MET-VEGFR2 interaction

To determine whether up-regulation of VEGFR2 by MET affects the sensitivity of tumor cells to VEGF, we explored the effect of VEGF on intracellular signal transduction. Interestingly, we found that VEGF plays a similar role to HGF in promoting MET phosphorylation. We also observed that VEGF induced activation of its downstream targets, ERK and AKT, in HCC827GR and PC-9GR cells with high MET expression, and the level of alterations were relatively weak in parental cells with low MET expression (Fig. 3A and B). Moreover, the above effect was significantly attenuated by blocking VEGFR2 with apatinib or vandetanib, and the expression of total MET protein remained unaffected (Fig. 3C). Next, we used co-immunoprecipitation (CoIP) to identify the interaction between MET and VEGFR2. PC-9 cells stably expressing Flag (PC-9/Vec) or Flag-MET(PC-9/MET) were harvested for immunoprecipitation using anti-IgG or anti-Flag antibodies and then analyzed by western blotting. We found that VEGFR2 interacted with Flag-MET, but not with Flag alone. This result indicated that there was a constitutive association between MET and VEGFR2 (Fig. 3D). We further found that, upon VEGF treatment, the presence of MET in the VEGFR2-immunoprecipitates increased, following phosphorylated MET significantly increased, which could be blocked in the presence of apatinib (Fig. 3E). These data independently supported a mechanism wherein VEGFR2 is constitutively associated with MET, and further recruits MET and facilitates its activation upon VEGF binding.

### Combined inhibition of MET and VEGF overcomes resistance to EGFR TKIs driven by overactivated MET in vitro

**Fig. 4:**
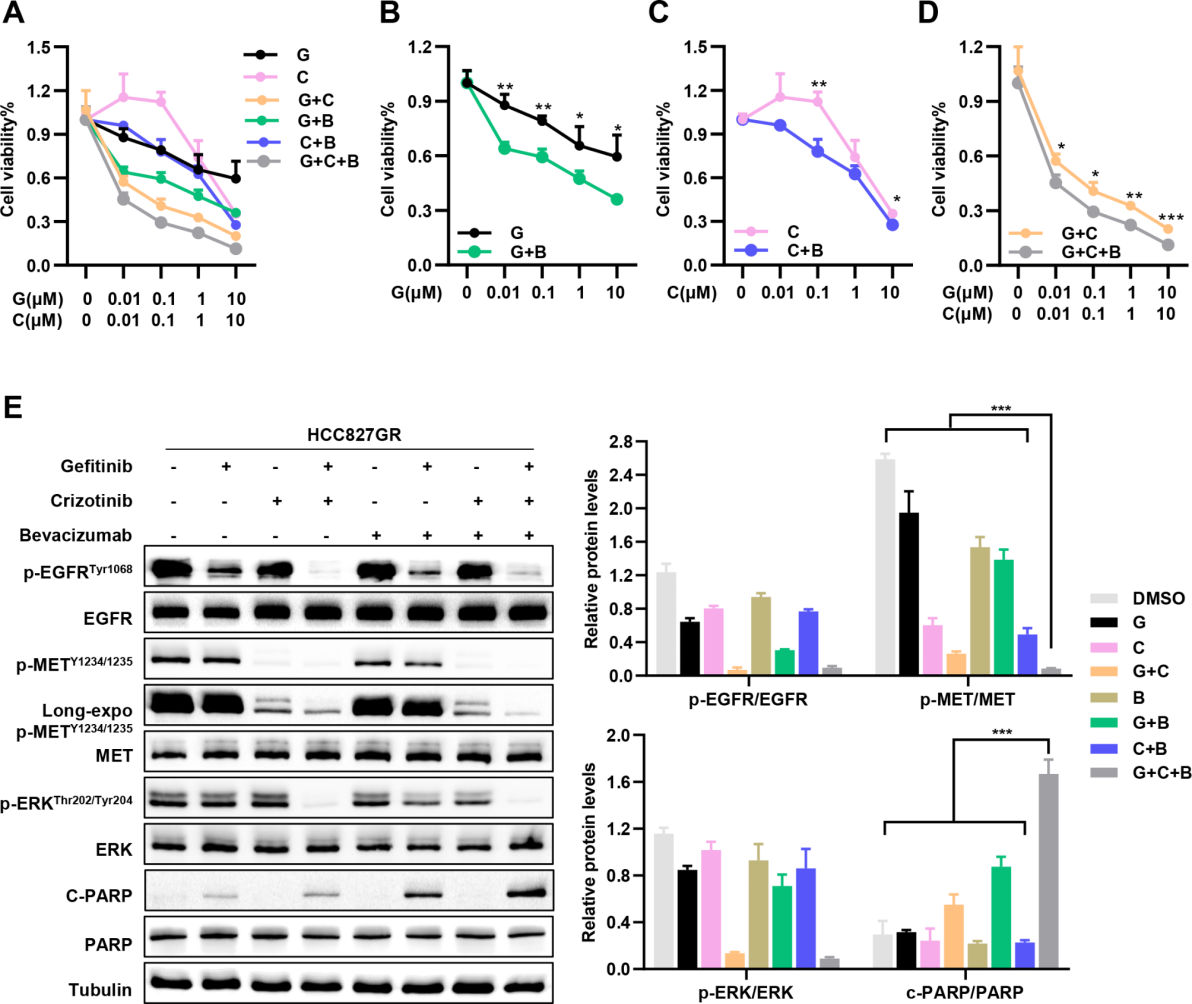
MET-Driven resistance to EGFR TKIs may be overcome by added using of the crizotinib and bevacizumab in *EGFR*-mutated NSCLC (**A-D**) HCC827GR cells were incubated with gefitinib, crizotinib, bevacizumab (200 µg/ml) or various combinations. Cell viability relative to untreated controls was measured by CCK-8 assays after 48 h. (**E**) Western blotting analysis of the expression of the indicated proteins and their phosphorylation levels in HCC827GR cells upon treatment with gefitinib, crizotinib, bevacizumab or various combinations. *P < 0.05, **P < 0.01, ***P < 0.001 (Student’s *t* test.). Graphs show mean ± SD

We further investigated the potential value of combined inhibition of MET and VEGF signaling to block the positive feedback loop in improving gefitinib sensitivity in drug-resistant NSCLC cells with *EGFR* mutation and abnormal MET activation. First, we combined crizotinib, bevacizumab, and gefitinib in the subsequent experiments. We found that addition of the VEGF or/and MET inhibitor was able to restore the sensitivity to gefitinib in MET transduced cells. Most importantly, the triple combination of gefitinib, crizotinib, and bevacizumab resulted in a significantly higher inhibition of cell growth relative to any single- or double-drug treatment over 48 h (Fig. 4A-D). Subsequently, this triple inhibition led to a greater decrease in MET and ERK signaling activity and significantly enhanced C-PARP expression in HCC827GR cells (Fig. 4E). These findings highlight the potential to prospectively identify treatment *EGFR*-mutant lung cancer patients with aberrant MET activation who may benefit from this three-drug combination therapy.

### Combined inhibition of MET and VEGF enhances the anti-tumor activity of EGFR TKIs in vivo

**Fig. 5:**
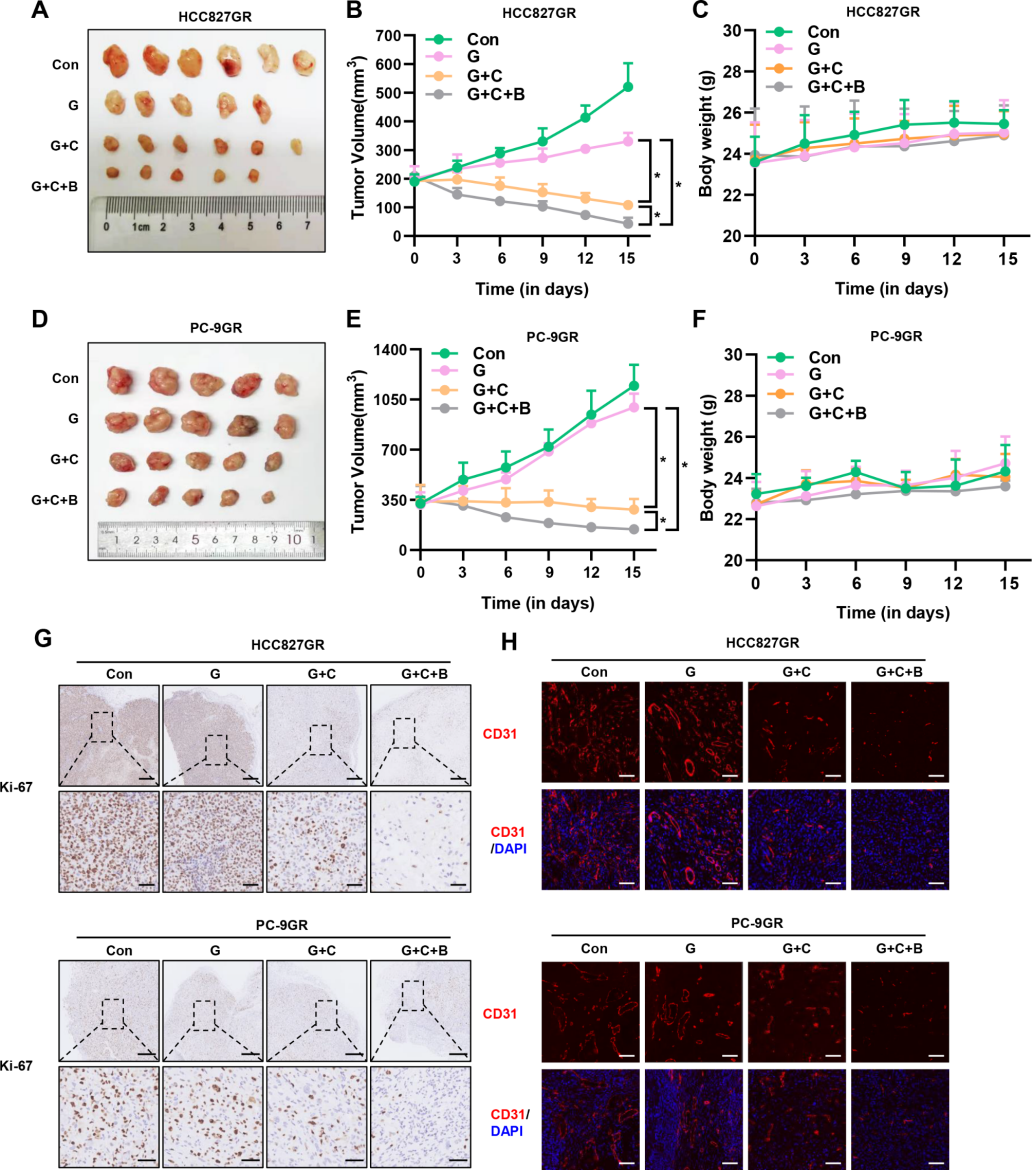
Triple inhibition of EGFR, MET, and VEGF suppresses growth and angiogenesis in HCC827GR and PC-9GR tumors in vivo (**A**) (**D**)NCG mice bearing HCC827GR or PC-9GR tumors (>200 mm^3^ in size) were administered gefitinib and/or crizotinib orally once daily and/or bevacizumab intraperitoneally each five days. Representative tumor xenografts of each group. (**B**) (**E**) Tumor growth curve in each group. (**C**) (**F**) Body weight of each group. (**G**) Tumor proliferating cells were determined by Ki-67 immunohistochemical staining. (**F**) Tumor vessels were determined by CD31 immunofluorescence staining. *P < 0.05, **P < 0.01, ***P < 0.001 (one-way ANOVA). Graphs show mean ± SD

**Fig. 6:**
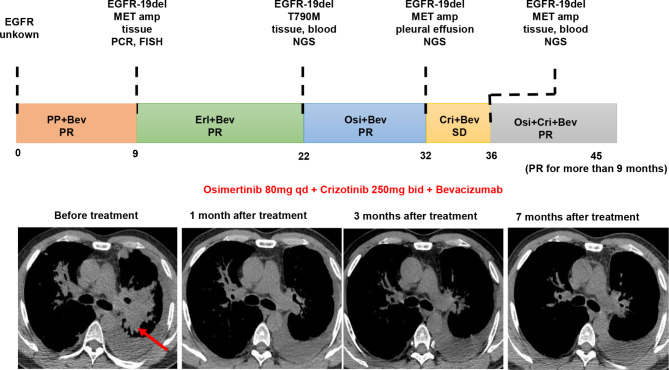
A lung adenocarcinoma with concomitant *EGFR* and de novo *MET* amplification response well to combination of TKI and bevacizumab The patient clinical history is summarized in Fig. 6. 44-year-old male stage IV lung adenocarcinoma with left lung tumor was detected harboring of *EGFR-19del* and *MET* amplification using PCR and FISH. The patient was treated with erlotinib + bevacizumab and achieved partial response (PR) with a PFS with 13 months. After PD, NGS performed on both tissue and plasma biopsies revealed that the patients obtained first-generation resistant mutation *EGFR-T790M*, concomitant with *EGFR-19del*. The patient was treated with osimertinib + bevacizumab and achieved PR. He developed PD again with a PFS of 10.2 months, and repeated biopsies sequencing identified concomitant *EGFR-19del* and *MET* amplification. Then, the patient was treated with crizotinib + bevacizumab and the best curative effect was stable disease. Four months later, he developed PD and the third biopsy still revealed positive *EGFR-19del* and *MET* amplification. The patient received osimertinib + crizotinib + bevacizumab and he achieved PR one month after treatment initiation and the PFS is more than nine months

To further investigate the potential effects of the combination of crizotinib and/or bevacizumab with EGFR TKIs in vivo, we generated subcutaneous tumors by injecting HCC827GR or PC-9GR cells (5 × 10^6^ cells/mouse) into highly immunodeficient NCG mice. Subsequently, mice bearing established subcutaneous tumors (tumor volume >200 mm^3^) were treated with various combination of gefitinib, crizotinib, and bevacizumab as shown in Supplemental Figure S3. After 6 h of the last treatment, the mice were sacrificed and photographed (Fig. 5A and D). Tumor growth curves over time are shown in Fig. 5B and E. There was no significant difference in body weight between each group, and thus the treatments were feasible (Fig. 5C and F). We could find that the tumor growth of parental HCC827 and PC-9 tumor bearing mice treated with gefitinib was significantly inhibited compared with saline treatment (Supplemental Figure S3B, S3C). However, gefitinib alone has limited inhibitory effect on the growth of HCC827GR and PC-9GR xenografts while gefitinib plus crizotinib inhibited tumor growth markedly. Most importantly, dual inhibition of MET and VEGF increased the anti-tumor effect of gefitinib in HCC827GR and PC-9GR tumors dramatically. Consistent with tumor volume data, tumor tissue samples from the triple combination group expressed lower Ki-67 protein levels and lesser degree of vascularization than any other group (Fig. 5G and H) while the level of apoptosis was higher (Supplemental Figure S4). Additionally, in the treatment of a lung adenocarcinoma in our center, we also established the feasibility of such combination therapy after EGFR TKI resistance. This patient we presented harboring concomitant *EGFR* mutation and de novo *MET* amplification obtained favorable response to combinatorial therapy of TKI and bevacizumab (Fig. 6).

## Discussion

Aberrant MET activation in NSCLC, which is associated with poor prognosis, reportedly mediates primary and acquired resistance to EGFR TKIs in *EGFR*-mutant NSCLC [33]. Previous preclinical studies have indicated that combining EGFR TKIs and MET inhibition may be necessary to overcome resistance caused by concurrent aberrant MET activation. However, clinical reports regarding successful combination of EGFR and MET inhibitors in *EGFR*-mutated/MET-aberrant NSCLC patients are limited, and data from various clinical trials have not been uniformly conclusive, the effective therapeutic strategy remains to be determined. Consistent with previous reports, our results show that lower concentrations of gefitinib were sufficient to inhibit the phosphorylation of AKT and ERK in parent HCC827 cells and PC-9 cells. In contrast, resistant cells maintained the phosphorylation levels of MET and downstream signaling AKT and ERK at higher concentrations of gefitinib, suggesting that the MET bypass compensates for continued activation of downstream signaling.

In our study, we assessed the effect of MET overactivation on the mRNA expression of *VEGFR1* and *VEGFR2* (VEGFRs) and their co-receptors *NRP1* and *NRP2* (NRPs). We found that increased MET activity in NSCLC cells up-regulated *VEGFR2*, but had no effect on *VEGFR1* and *NRPs* levels. Furthermore, we found that shRNA-mediated *MET* silencing or pharmacological inhibition of MET led to significantly decreased VEGFR2 levels. Similarly, previous studies also found a positive regulation of VEGFR2 by activated MET signaling in endothelial cells, primary Schwann cells (SCs) and Vestibular schwannoma cells (VS) [28]. Moreover, our results demonstrated that MET regulated the expression of VEGFR2 in a MAPK/ERK-dependent manner. According to previous data, ERK could translocate to the nucleus and promote ETS binding to the FOX: ETS composite site in the first intron enhancer of *VEGFR2* thereby enhancing its transcription and expression [34]. VEGFR2 expression has been reported to be positively correlated with the effect of anti-VEGF therapy, further suggesting that NSCLC patients with MET overactivation may benefit from this treatment [35].

Previous studies have shown that VEGF may regulate the MET signaling pathway either positively or negatively via different mechanisms. In endothelial cells, siRNA-mediated knockdown of VEGFA or decreasing VEGF-A levels by pharmacological means, such as bevacizumab, led to a decrease in MET expression [36]. NRP1 and MET are physically associated on the plasma membrane of prostate cancer cells, and in response to VEGF stimulation, their interaction significantly facilitates further recruitment and activation of MET [37]. In contrast, an autocrine VEGF/VEGFR2 loop in glioblastoma multiforme cells negatively affected MET activity through recruitment of the phosphatase PTP1B to the VEGFR2/MET complex [38]. In our study, we observed that VEGF facilitated MET phosphorylation and activated several intracellular signaling pathways in *EGFR*-mutant NSCLC cells with high MET expression, thus promoting cell proliferation. Conversely, bevacizumab significantly reduced the phosphorylation level of MET and blocked VEGF enhanced proliferation in these cells. Intriguingly, we presented evidence that physical interaction between VEGFR2 and MET may enhance the phosphorylation of MET. When selectively inhibiting VEGFR2, the interaction between VEGFR2 and MET induced by VEGF was reduced. Based on the above results, our experiment suggested for the first time that there was a positive feedback regulation between MET and VEGF/VEGFR2 in NSCLC cells. However, the structural and molecular basis for the interaction between MET and VEGFR2 in NSCLC needs to be further investigated.

In addition, our results indicated that additional anti-VEGF therapy led to more decrease in ERK and AKT phosphorylation than gefitinib and/or crizotinib alone. Most importantly, although pre-existent *MET* amplification has been reported as a determinant of shorter time on EGFR TKIs [39], the patients we presented in Fig. 6 harboring concomitant *EGFR* mutation and de novo *MET* amplification obtained favorable response to combinatorial therapy of TKI and bevacizumab. Notably, the patient harboring dual *EGFR*-*MET* alterations in this study achieved 13 months PFS upon treatment with bevacizumab plus erlotinib, which was similar with those patients with *EGFR*-mutation only. These results suggested that some patients with *EGFR* mutations associated with aberrant MET activation might have been benefited from first-line treatment of EGFR TKI combined with bevacizumab.

Moreover, our experiments suggested that triple therapy with gefitinib, crizotinib and bevacizumab exerted the most significant inhibitory effect on downstream signaling and cell proliferation in *EGFR*-mutant NSCLC cells with concomitant aberrant MET activation. Meanwhile, triple inhibition of EGFR, MET, and VEGF effectively inhibited tumor growth and angiogenesis of MET-driven EGFR-TKI resistant tumors. The clinical case we presented also substantiated the feasibility of this combination therapy. Two previous pre-clinical studies by Professor Seiji Yano indicated that HGF induced resistance to EGFR TKIs by activating the MET/PI3K pathway [40, 41]. These studies suggested that dual inhibition of HGF/MET and VEGF signaling pathway would be therapeutically useful for controlling HGF-induced EGFR-TKI-resistant lung cancer. Although HGF tended to induce a series of biological changes by activating MET, resistance to EGFR TKIs caused by abnormal MET activation was not necessarily accompanied by the changes of HGF [42]. Therefore, MET activation status appears to be a better biomarker than HGF expression, and clinical trials are needed to evaluate the efficacy and safety of the triple therapy of MET and VEGF inhibition combined with EGFR TKIs in *EGFR*-mutant lung cancer patients who acquired EGFR-TKI resistance due to MET overactivation.

Previous studies have reported that blocking VEGF/VEGFRs signaling may lead to hypoxia in the tumor microenvironment by inhibiting angiogenesis, resulting in activation of HGF/MET axis, which conferred drug resistance and increased local invasion and distant metastasis [43, 44]. A study of NSCLC found that tumors treated with short-term VEGFR TKIs had reduced levels of MET compared with sensitive tumors, while MET was up-regulated in tumors that progressed on long-term treatment, thus making the tumor more refractory to treatment after the initial response stage [45]. Anyway, combined MET and VEGF inhibition may be valuable in overcoming not only EGFR TKIs resistance, but also angiogenesis inhibitor resistance.

Finally, it is worth noting that, in addition to being a well-known mechanism of resistance to EGFR-TKIs, increasing research has also emphasized the significant role of *MET* amplification or abnormal activation in driving resistance to targeted therapies in other driver gene-positive NSCLC patients (including *ALK*-, *RET*-, *ROS*-1-fusion-positive, and *KRAS G12C*-mutant lung cancers) [46, 47]. Remarkably, the prevalence of this phenomenon is similar across these groups, with an overall incidence of approximately 15%. Therefore, TKI resistance caused by MET amplification or abnormal activation deserves more attention in this field. There is a need to prospectively identify and utilize effective and safe combination therapies for these patients. The positive feedback loop between MET and VEGF/VEGFR2 may serve as a potential molecular marker for combination therapy in advanced NSCLC patients after resistance develops, and combined inhibition of MET and VEGF signaling pathways may further enhance the efficacy of the corresponding TKIs and overcome resistance.

## Conclusions

**Fig. 7:**
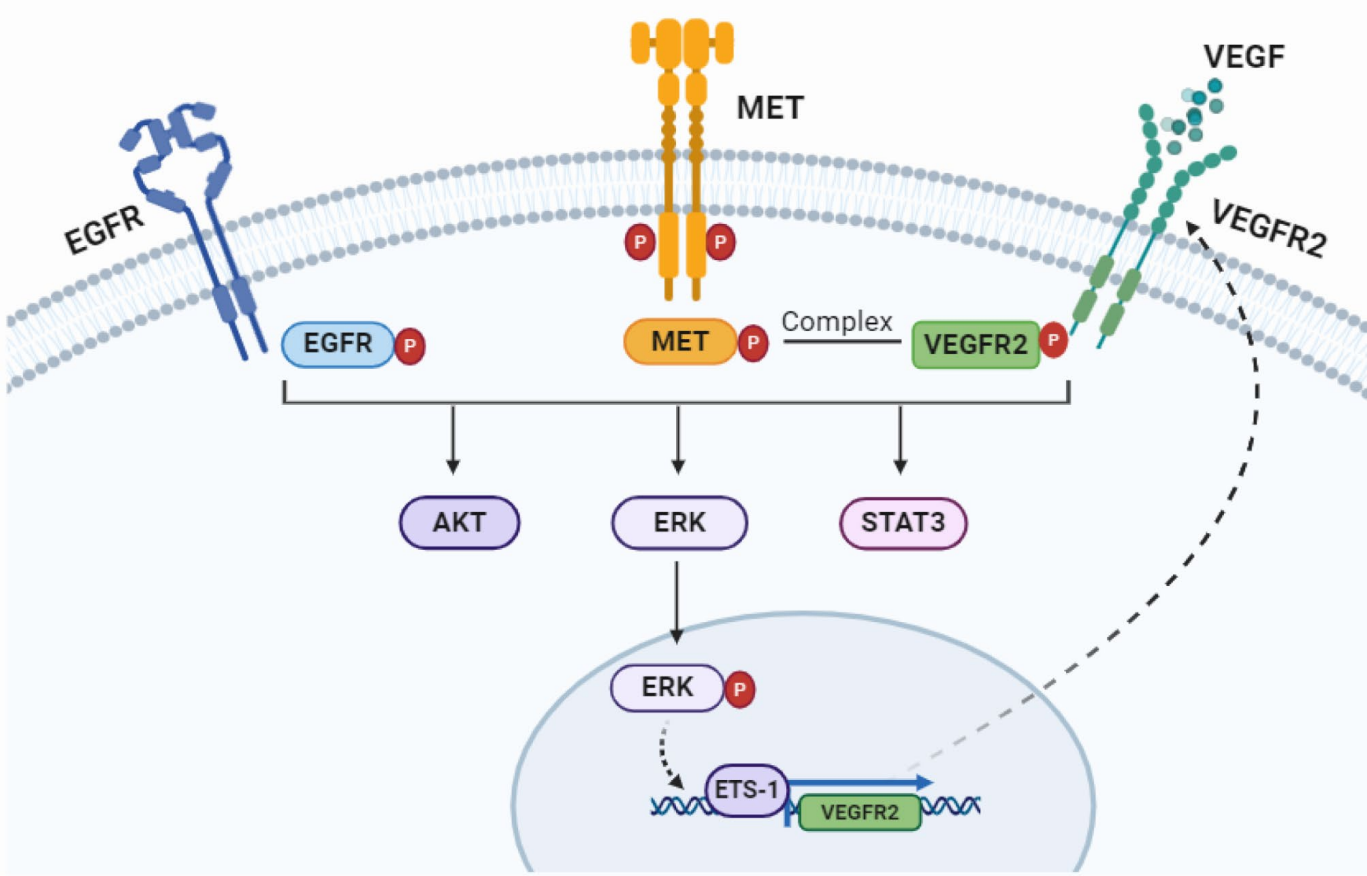
Schematic diagram showing the mechanism of the interaction between MET and VEGF/VEGFR2 signaling in *EGFR*-mutant NSCLC cells with MET overactivation

Our findings uncover a unique cross-regulation between MET and VEGF/VEGFR2-two prominent signaling pathways for the first time. Specifically, MET up-regulated VEGFR2 expression in a MAPK/ERK/ETS1-dependent manner, while VEGF promoted physical interaction between VEGFR2 and MET, thus forming a positive feedback loop in NSCLC. Combined inhibition of MET and VEGF enhanced antitumor activity of EGFR TKIs in *EGFR*/*MET* co-altered NSCLC by disrupting this loop in a synergistic manner, thereby weakening downstream signal transduction, inhibiting proliferation, and impairing angiogenesis (Fig. 7). These findings presented a promising strategy for *EGFR*-mutant NSCLC with concomitant aberrant MET activation. In addition, biomarkers for detecting the activities of MET and VEGFR2 may be necessary for the optimal use of inhibitors for MET and VEGF/VEGFRs.

## Electronic supplementary material

Below is the link to the electronic supplementary material.

Supplemental Figure S1

Supplemental Figure S2

Supplemental Figure S3

Supplemental Figure S4

Supplementary Material 5

Supplementary Material 6

Supplementary Material 7

## Data Availability

No datasets were generated or analysed during the current study.

## Abbreviations

MET: Mesenchymal-epithelial transition factor
VEGF: Vascular endothelial growth factor
EGFR: Epidermal growth factor receptor
TKI: Tyrosine kinase inhibitor
NSCLC: Non-small cell lung cancer
VEGFR2: Vascular endothelial growth factor receptor 2
ETS1: ETS proto-oncogene 1
HGFR: Hepatocyte growth factor receptor
HGF: Hepatocyte growth factor
PFS: Progression-free survival
CoIP: Co-immunoprecipitation
NGS: Next generation sequencing
FISH: Fluorescence in situ hybridization
KDR: Kinase domain receptor
TCGA: The cancer genome atlas
CCK8: Cell counting kit-8
NCG: NOD-*Prkdc*^*em26Cd52*^*il2rg*^*em26Cd22*^/Nju
IHC: Immunohistochemistry
SD: Standard deviation
PR: Partial response
PD: Progressive disease
