# Supplementary figures and images for: Combined inhibition of MET and VEGF enhances therapeutic efficacy of EGFR TKIs in EGFR-mutant non-small cell lung cancer with concomitant aberrant MET activation
Source: Exp Hematol Oncol. 2024 Oct 1;13:97. doi: 10.1186/s40164-024-00565-9 (PMC11443824; doi:10.1186/s40164-024-00565-9)

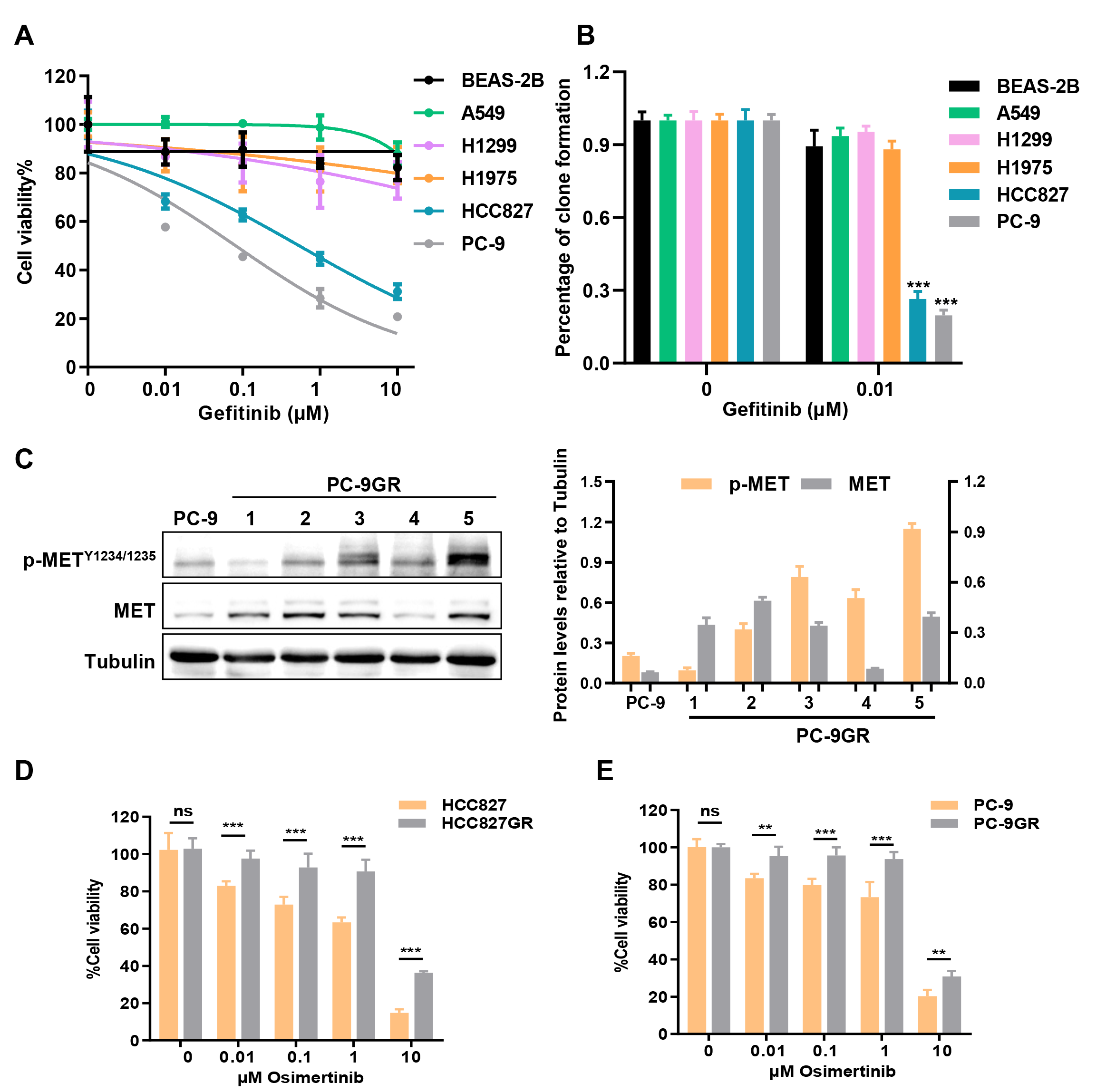

Supplement: Supplementary file 1 — Supplemental Figure S1 [file 40164_2024_565_MOESM1_ESM.png]

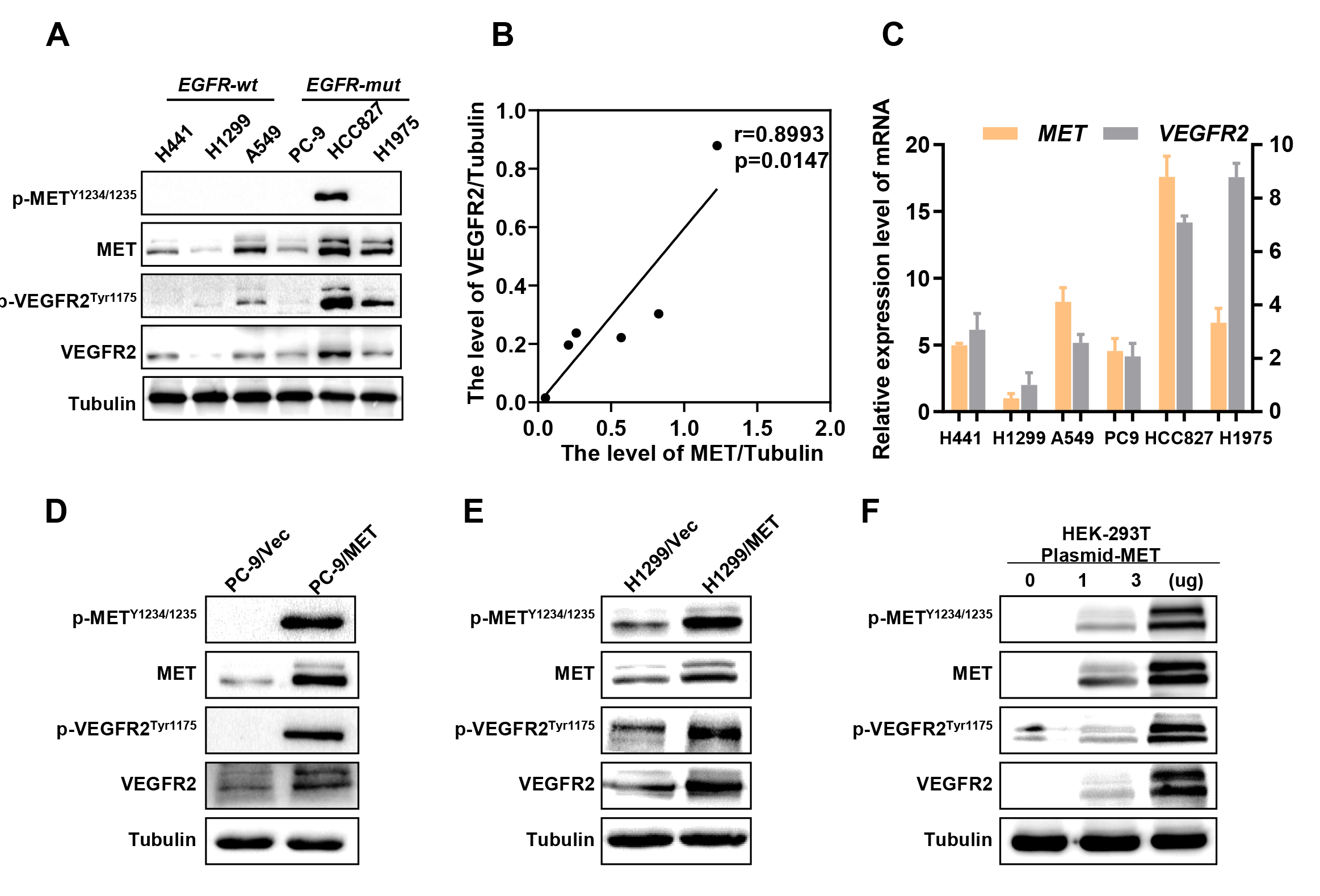

Supplement: Supplementary file 2 — Supplemental Figure S2 [file 40164_2024_565_MOESM2_ESM.png]

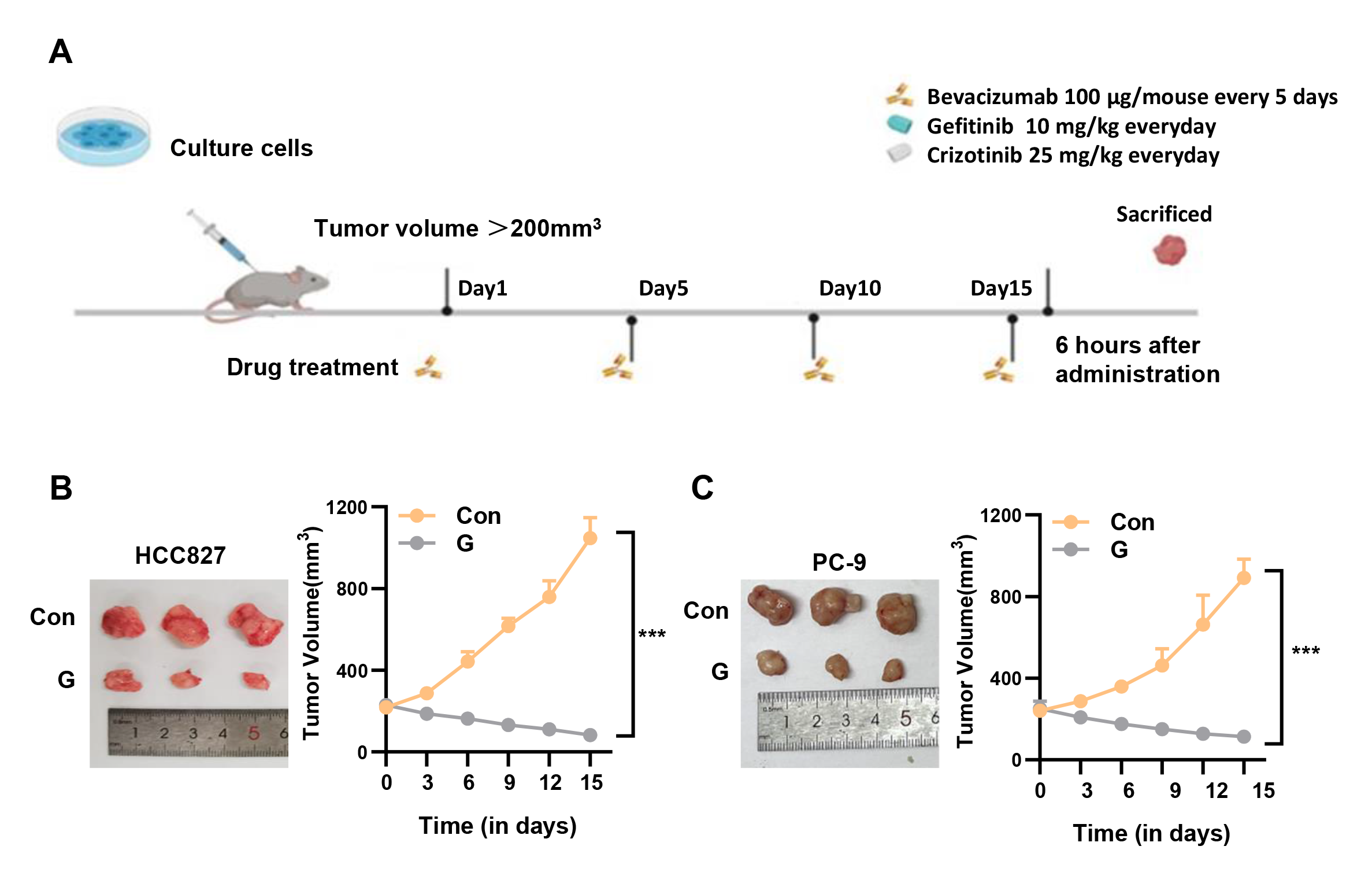

Supplement: Supplementary file 3 — Supplemental Figure S3 [file 40164_2024_565_MOESM3_ESM.png]

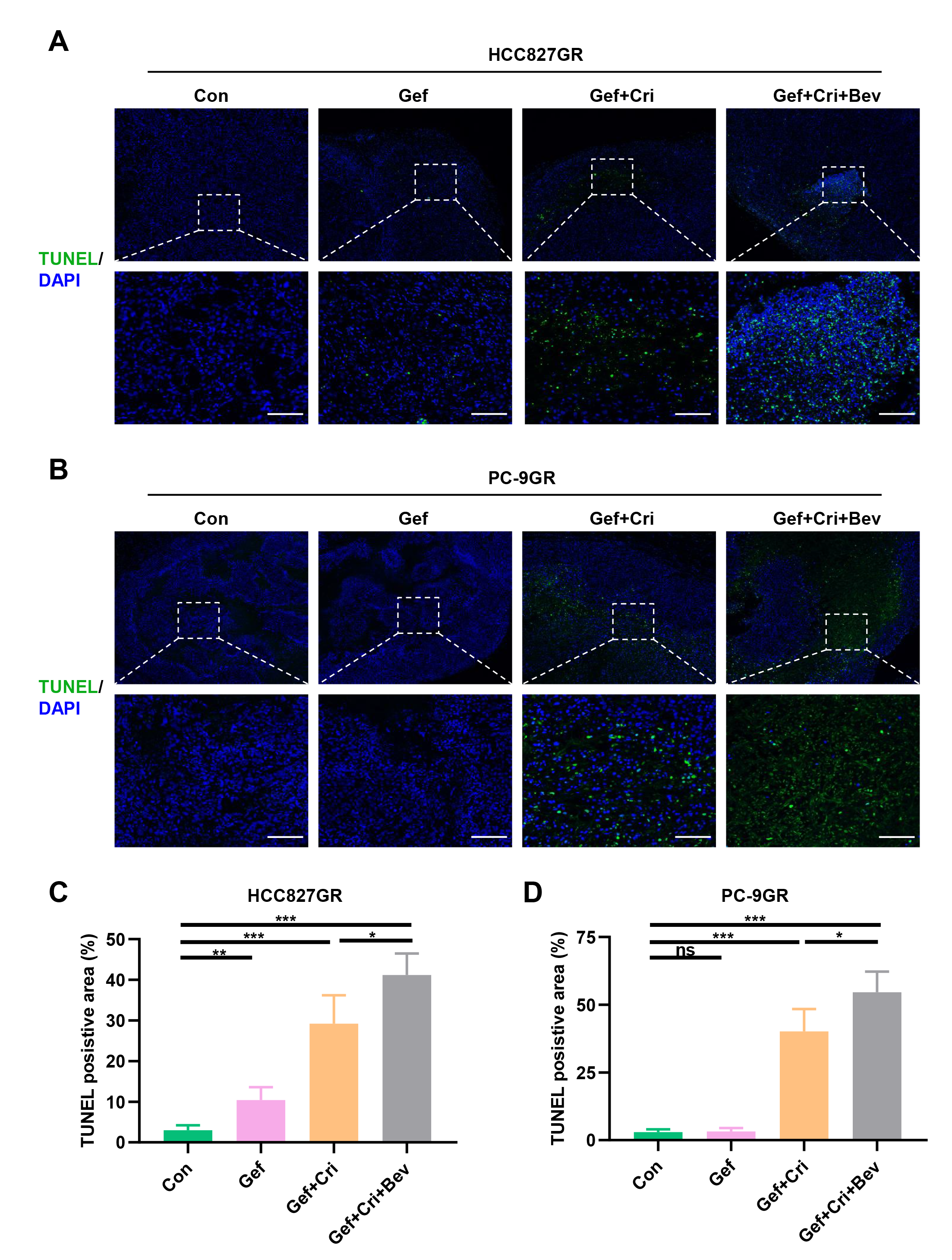

Supplement: Supplementary file 4 — Supplemental Figure S4 [file 40164_2024_565_MOESM4_ESM.png]
