# Supplementary material for: Combined inhibition of MET and VEGF enhances therapeutic efficacy of EGFR TKIs in EGFR-mutant non-small cell lung cancer with concomitant aberrant MET activation
Source: Exp Hematol Oncol. 2024 Oct 1;13:97. doi: 10.1186/s40164-024-00565-9 (PMC11443824; doi:10.1186/s40164-024-00565-9)
Supplement: Supplementary file 7 — Supplementary Material 7 [file 40164_2024_565_MOESM7_ESM.doc]

Supplementary Information

**Supplemental Figure S1. Establishment of gefitinib resistant NSCLC cell lines with concomitant aberrant MET activation.** (A) Survival curves of different cell lines treated with gefitinib. (B) Clonal formation of indicated cell lines after 14 days treated with 0.01 μM gefitinib. (C) Western blot analysis of MET expression and its phosphorylation level in parent PC-9 cells and PC-9GR resistant clones. (D) (E) Cells treated with increasing concentrations of osimertinib. Cell viability relative to untreated controls was measured by CCK-8 assays after 48 h.

**Supplemental Figure S2. MET was positively correlated with VEGFR2 in NSCLC cells.** (A) Western blot analysis of the protein level of MET and VEGFR2 in six NSCLC cell lines. (B) Quantitative and correlation analysis of protein expression of MET and VEGFR2 in six NSCLC cell lines. (C) qPCR mRNA analysis of the mRNA level of MET and VEGFR2 in six NSCLC cell lines. (D) Correlation analysis of MET and VEGFR2 in 197 kinds of lung cancer cells from cBioPortal database. (E) Correlation analysis of MET and VEGFR2 in 1129 patients with lung cancer in TCGA database.

**Supplemental Figure S3.** (A) Flow chart of animal experiment operation. (B) (C) Tumor growth curve in HCC827 tumors and PC-9 tumors.

**Supplemental Figure S4.** Tumor cell apoptosis was detected by TUNEL staining.

**Supplemental Table S1.** The primary antibodies used for experiments in this study.

**Supplemental Table S2.** The sequences of primers used for experiments in this study.
